# Gut Dysbiosis in Children with Cystic Fibrosis: Development, Features and the Role of Gut–Lung Axis on Disease Progression

**DOI:** 10.3390/microorganisms11010009

**Published:** 2022-12-20

**Authors:** Ilaria Testa, Oliviero Crescenzi, Susanna Esposito

**Affiliations:** 1 Respiratory Unit, Great Ormond Street Hospital for Children NHS Foundation Trust, London WC1N 1LE, UK; 2 Department of Anaesthesia, Hammersmith Hospital, Imperial College Healthcare NHS Trust, London WC1N 1LE, UK; 3 Paediatric Clinic, Department of Medicine and Surgery, University of Parma, 43126 Parma, Italy

**Keywords:** cystic fibrosis, gut dysbiosis, gut–lung axis, microbiota, probiotics

## Abstract

Cystic fibrosis (CF) is the most common autosomal recessive disease among Caucasians. Over the last 20 years, culture-independent analysis, including next-generation sequencing, has paired with culture-based microbiology, offering deeper insight into CF lung and gut microbiota. The aim of this review is to analyse the features of gut microbiota in patients with CF and its possible role in the progression of the disease, establishing the basis for a potential role in microbe-based therapies. The literature analysis showed that the gut environment in CF patients has unique features due to the characteristics of the disease, such as decreased bicarbonate secretion, increased luminal viscosity, and an acidic small intestinal environment, which, due to the treatment, includes regular antibiotic use or a high-energy and fat-dense diet. As a result, the gut microbial composition appears altered, with reduced richness and diversity. Moreover, the population of pro-inflammatory bacteria is higher, while immunomodulatory genera, such as *Bacteroides* and *Bifidobacterium*, are scarcer. The imbalanced gut microbial population has a potential role in the development of systemic inflammation and may influence clinical outcomes, such as respiratory exacerbations, spirometry results, and overall growth. Although a better understanding of the pathophysiology behind the gut–lung axis is needed, these findings support the rationale for considering gut microbiota manipulation as a possible intervention to regulate the severity and progression of the disease.

## 1. Introduction

Cystic fibrosis (CF) is the most common autosomal recessive disease among Caucasians, affecting nearly 50,000 individuals in Europe and more than 85,000 individuals worldwide [1,2]. CF is associated with mutations in the gene coding for the cystic fibrosis transmembrane conductance regulator (CFTR) protein [3]. The CFTR protein functions on the apical surface of epithelial cells in the airways, pancreas, intestines, and hepatobiliary tree as an anion-selective ion channel (mainly chloride and bicarbonate) and thus contributes to epithelial fluid secretion and intra-luminal mucus hydration [4].

The lungs are the most seriously affected organ in CF, and respiratory diseases are the main cause of reduced life expectancy. As a result, the respiratory tract has received the greatest research attention. However, the CFTR protein is located throughout the body on the apical layer of the epithelial cells, resulting in multiple morbidities, including altered gastrointestinal functioning [4]. Patients with CF have reduced bicarbonate secretion from the pancreas, intestine, and biliary tree as part of the primary *CFTR* defect. A lack of bicarbonate results in increased luminal viscosity due to the formation of inspissated mucus in the intestinal tract as well as in a more acidic small intestinal environment. These features, along with the regular use of antibiotics due to recurrent pulmonary infections, the increased load of malabsorbed luminal contents, a high-energy and fat-dense diet, the use of a pancreatic enzyme, and impaired innate immunity may contribute to the development of microbial gut dysbiosis that has been observed in patients suffering from CF [5].

Over the last 20 years, culture-independent analysis, including next-generation sequencing, has paired with culture-based microbiology, offering deeper insight into CF lung and gut microbiota. In particular, 16S ribosomal RNA gene sequencing has allowed for the identification and relative enumeration of the bacterial taxa within a clinical sample in a way that would have been unachievable using culture-based approaches [6]. The role of gut microbiota in driving a healthy immune response is generally acknowledged, and several studies have addressed its role in various chronic respiratory conditions, such as asthma, chronic obstructive pulmonary disease (COPD), and CF [7,8]. The aim of this review is to analyse the features of gut microbiota in patients with CF and its possible role in the progression of the disease, establishing the basis for a potential role in microbe-based therapies.

## 2. Gut Microbiome in CF: Development and Influences

Microbiota acquisition begins in the uterus and progresses during childhood. Its composition is influenced by many factors, including delivery method, maternal physical contact, feeding, and exposure to antibiotics [9,10,11]. Those factors, which are well-known influences on gut microbiota composition in the general population, also play a role in the CF population along with CFTR dysfunction (Table 1).

Age is a further key point in gut microbiome development. In the general population, the greatest inter-individual variability in gut microbiota occurs within the first 3 years of life [23,24]. After that, it resembles that of the adult, remaining relatively stable with few further perturbations. Loman et al. noted that age is the strongest predictive factor of overall faecal microbial composition in CF children as well [12]. Dietary factors, the introduction of solid food, and the subsequent different substrate availability are the main drivers for such changes.

### 2.1. CFTR Dysfunction

CFTR dysfunction actively modulates and selects the gut microbiome, as demonstrated in a study performed on mice in the absence of confounding factors, such as diet or antibiotic treatment [13].

CFTR gene variants have been investigated as a possible influencer of gut microbiota in CF patients who are not on antibiotics: *Escherichia coli* and *Eubacterium biforme* species were found to be prevalent in patients with F508del mutations, especially in the homozygous state and in more severe CF patients, while beneficial species, such as *Faecalibacterium prausnitzii, Bifidobacterium* spp., and *Eubacterium limosum*, were reduced [15]. Burke et al. found no significant differences in species richness or microbial diversity between the CF cohort with class 1–3 mutations, which are considered the most severe, and CF individuals with mutations from other classes [14]. However, differences were noted at a genus level and at a family level, with *Enterococcaceae* being significantly higher and *Ruminococcaceae* significantly lower than those with less severe mutations [14].

More recently, new studies have been investigating the effect of the initiation of CF modulators on CF microbiota. Ooi et al. noted no significant difference in alpha and beta diversities in 16 CF patients (eight children and eight adults; two are pancreatic sufficient) 6 months after starting ivacaftor [16]. However, they noted a significant reduction in faecal calprotectin and an increase in the relative abundance of the bacterial genus *Akkermansia*, which resides in the intestinal mucus layer, stimulates host mucosal anti-inflammatory pathways and improves epithelial barrier integrity. An additional study of 14 adults suffering from CF and pancreatic insufficiency confirmed no significant change in gut microbiota diversity and richness after a year of treatment with ivacaftor but did not confirm a reduction in faecal calprotectin [17].

Overall, CFTR dysfunction and CF disease severity contribute to shaping the composition of gut microbiota in CF patients. Very limited data are available on the potential influence of CFTR modulators investigating, in particular, the possible impact of a potentiator. According to the evidence available, there is a possible impact of the ivacaftor on mucus layer commensals and on gut inflammation.

### 2.2. Delivery Method

Delivery method is a factor known to influence gut microbiota composition in the general population. Few studies investigating its role in CF patients are available, and there is no unified evidence to date.

Birth has been analysed as a possible influencing factor in CF patients by Loman et al. [12]. Using 16S RNA gene sequencing, they analysed faecal samples from a group of children between 3 months and 5 years of age, all suffering from CF, who have at least one copy of the Phe508del mutation and are pancreatic insufficient. Their study noted that birth via Caesarean section was associated with higher alpha diversity than vaginal birth. Additionally, a single genus, *Turicibacter*, was higher in children born via Caesarean section and undetected in all vaginally born children [25]. This taxon was reported as putatively pro-inflammatory in animal models [26]. These results have not been confirmed by a more recent study performed in 2019 by Antosca et al. [18] that compared stool samples collected from 21 infants with CF during their first year of life with samples collected from 409 infants without CF from the New Hampshire Birth Cohort Study. The delivery mode had been previously shown to significantly affect the gut microbiota of infants in the New Hampshire Birth Cohort Study, yet this effect was not observed in the CF cohort, even after an adjustment for antibiotic use at delivery [18].

### 2.3. Breastfeeding

Breastfeeding and its influence on the gut microbiome is another factor that has been investigated. Loman et al. compared children affected by CF who were exclusively formula-fed, breastfed, and mixed formula- and breastfed; faecal samples from the three subgroups were found to have similar alpha and beta diversities [12]. However, they noted a higher relative abundance of the genus *Lactococcus* in children who were exclusively formula-fed. Although the analysed studies involved a small number of patients, it is interesting to note that factors such as the delivery method or feeding (breastfeeding or formula), which are well-known influences on the gut microbiota composition in the general population, seem not to affect it significantly in children with CF. Madan et al. followed up with seven CF patients from birth to 9–21 months of age and noted non-significant overall diversities in the gut microbial populations between breastfed and formula-fed infants; however, they did note the significant effects of breast milk exposure on respiratory tract diversity [19]. A possible explanation for the lack of influence of breastfeeding on gut composition in CF children was proposed by Vernocchi et al.: gut microbiota composition in children with CF may be intrinsically linked to CFTR impairment and minimally influenced by other external or internal factors that are usually involved [20].

A limited number of studies have investigated the impact of breastfeeding on gut microbiota composition in children suffering from CF. However, considering what has been described above, there are no significant effects of breastfeeding on microbiota diversity, probably due to the major influence of CFTR impairment.

### 2.4. Antibiotic Treatment

The use of antibiotics and their role in driving the gut microbiome in CF patients is a major area for research considering the high frequency of their use as prophylaxis or treatment for respiratory tract infections as well as their effects on gut microbiota. Antibiotic therapies are known to reduce the diversity of intestinal microbiota and to alter the relative abundances of susceptible bacterial species in non-CF individuals [27]. Intestinal microbiota tends to return to normal in a few weeks after the treatment, yet some taxonomic changes may persist for a longer period of time [14]. Moreover, the human gut microbiota acts as a reservoir of resistance, and it is probable that the greater the exposure to antibiotics, the greater the pressure to select resistant microorganisms [28].

Numerous studies have tried to address the influence of antibiotic use on gut composition in CF patients. Vernocchi et al. analysed faecal samples from 31 children suffering from CF between 1 and 6 years of age and compared them with healthy controls [20]. CF patients were classified based on chronic antibiotic regimen (no antibiotics, aerosol antibiotic therapy, or azithromycin plus aerosol) and on the requirement of a pulmonary exacerbation regimen. In their study, a single antibiotic therapy regimen did not significantly impact the alpha diversity of gut microbiota, while azithromycin plus aerosol antibiotic therapy worsened the alpha diversity as compared to healthy controls [20].

De Freitas et al. compared two subgroups of children/adolescents with CF, one requiring antibiotic therapy (CFAB) and one not (CFnAB). *Bifidobacterium* was the only microorganism analysed that was significantly lower in the CFAB group than in the CFnAB group. Their study also noted a positive correlation between body mass index (BMI), nutritional status, and Bifidobacterium in the CF group [21]. Many studies have already reported an antibiotic-induced decrease in the *Bifidobacterium* count in the general population. Duytschaever et al. confirmed this finding by comparing CF children and their siblings [29]. A lower count of *Bifidobacterium* in CF patients that received macrolides, higher levels of *Firmicutes*, and lower levels of *Bacteroides* were also noted in CF patients requiring more courses of intravenous (IV) antibiotics in a study performed on CF adults compared to the healthy population, although patients were investigated during a period of stability [14]. More recently, Kristensen et al. reported an independent association between antibiotic treatment (mainly co-trimoxazole) and lower alpha diversity in CF infants, a reduced abundance of *Bifidobacterium* and *Bacteroides*, and a higher abundance of *Enterococcus* [22]. *Bifidobacterium* has been related to a healthy gut, as it is involved in immune maturation, the production of vitamin B, antioxidants, and the production of short-chain fatty acids (SCFAs); the low abundance of *Bifidobacterium* and the higher abundance of *Enterococcus* may contribute to a pro-inflammatory profile [22].

Human gut microbiota act as a reservoir of antibiotic resistance in the general population [30]. Using shotgun metagenomic sequencing, Fouhy et al. analysed faecal samples from six CF patients during a period of clinical stability who had been exposed to oral, IV, inhaled, and long-term maintenance antibiotics in the 12 months prior to the sample collection and compared it with six non-CF controls [28]. They found a higher abundance of gene families and pathways involved in antibiotic resistance, including porin activity and penicillin-binding, in particular in *Lachnospiracheae, E. faecalis*, *Clostridium*, and *Bacteroides* [28]. A higher prevalence of amoxicillin and amoxicillin-clavulanic acid-resistant *Enterobacteriaceae* has been found in CF patients compared to their healthy siblings by Duytschaever et al. [31], and, more recently, higher proportions of aminoglycoside-resistant Gram-negative bacteria and extended-spectrum beta-lactamase (EBLS) *E. coli* were found in CF patients compared to healthy adults from another group [32].

Therefore, it is clear that the use of a combination of multiple antibiotics in CF patients reduces the gut composition diversity, causes a reduced abundance of immunomodulating genera, and increases the prevalence of the gene families and pathways involved in antibiotic resistance.

## 3. Gut Microbiome in Cystic Fibrosis: Composition and Features

CF patients are known to have imbalanced gut microbial compositions, with a higher degree of gut dysbiosis observed among patients with severe phenotypic expression and homozygous F508del mutations [15]. A microbial imbalance is characterized by a higher amount of pro-inflammatory microbiota, such as *Escherichia* and *Enterococcus*, than immunomodulatory genera, such as *Bacteroides* and *Bifidobacterium* [33]. The abundance of *Enterobacteriaceae*, particularly *Escherichia coli*, has been shown to be 10 times higher in CF compared to healthy controls [34].

**Figure 1:**
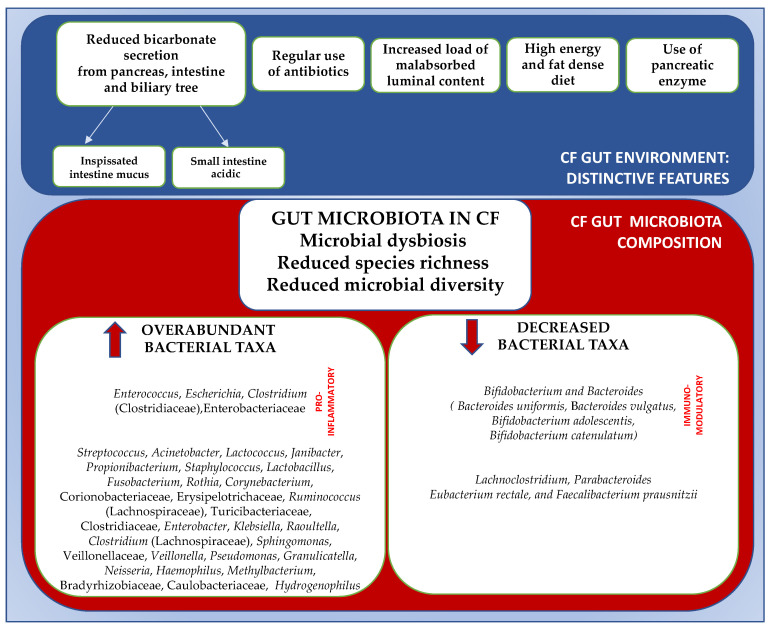
Gut distinctive features and gut microbial imbalance in CF patients. ↑, increased; ↓, decreased. Adapted from references [12,13,20,36].

Species richness is defined as the number of microbial species identified in an ecosystem. Overall, the gut microbiome in CF patients is characterized by the trend of lower species richness compared to the healthy population [12] and by an altered gut microbial balance known as dysbiosis [34,35] (Figure 1).

Nielsen et al. compared the microbial communities within the gastrointestinal tracts of children with and without CF (either pancreatic sufficient or insufficient) across a range of ages (0.87–17 years) [5]. They noted that gut microbial richness increases with age for both healthy and CF cohorts, but that it remains systemically lower in the CF cohort. Moreover, the microbial richness in the CF cohort during the teenage years does not even reach the same richness found in the CF cohort in infancy [5,36]. Lower gut microbiota richness in CF children, as compared to healthy controls matched by age and gender, has been confirmed by Coffey et al. [37] and by Duytschaever et al. in a comparison between CF children and their healthy siblings [29].

Microbial diversity takes both richness and evenness (relative abundance of each represented species) into consideration [33]. Paediatric CF patients are known to have a significantly lower α-diversity and a distinct beta diversity compared to healthy children [20]. Nielsen et al. noted also that the diversity of gut microbial communities increases with age in healthy children but not in children with CF. As a result, there is a progressive difference in the species diversity between those two populations, increasing with age [5]. Increased gut diversity has been repeatedly associated with health whilst decreased diversity has been associated with several inflammatory, metabolic, immune-mediated, and systemic diseases. These changes are potentially relevant, as the gastrointestinal microbiota of young children have been proposed as a determinant of respiratory and systemic disease progression [37]. Dysbiosis, reduced species richness and microbial diversity, as well as a higher pro-inflammatory than immunomodulating genera are the changes identified in children suffering from CF in multiple studies with potential influence on disease progression.

## 4. Gut Microbiota in Cystic Fibrosis: The Gut–lung Axis

The “gut–lung axis” is defined as the ability of the gut microbiota to influence the course or outcome of the underlying lung disease, and vice versa. This concept is well-described in other chronic respiratory diseases, such as asthma and COPD, where the gut microbiota has been suggested to influence lung health outcomes [38]. The interaction between the gut and the lungs is mainly based on the direct impact that the gut microbial community has on the immune system, but it also involves the passage of endotoxins, microbial metabolites, cytokines, and hormones into the bloodstream [39]. The gut microbiome can have a modulating effect on immune function. For example, *Bacteroides fragilis* modulates the Th type 1/2 (Th1/Th2) balance, and segmented filamentous bacteria directly stimulate Th17 cell differentiation, whereas *Clostridium* spp. induces Treg production [7]. Furthermore, metabolites such as SCFAs are involved in promoting recruitment, as well as in the maturation of the immune cells, which provide protection against an inflammatory response [40].

The crosstalk between the lungs and the gut in CF is particularly interesting: both sites are disrupted by CFTR loss-of-function and characterized by dysbiosis. Furthermore, this crosstalk and its possible immunomodulatory action are relevant in view of the known role of the pro-inflammatory cascade in CF lung disease [41,42]. This interaction and its effects have been noted in several studies (Table 2), although the exact mechanism of how the intestinal microbiome influences the immune response is not always fully understood.

Evidence of interaction between lung health and the gut microbiome was noted by Madan et al. during a follow-up with a small group of seven CF patients from birth to 9–21 months of age [19]. In this study, they noted a significant effect of breast milk exposure on respiratory tract diversity. Moreover, they noted that changes in diet also resulted in altered respiratory microflora. This finding confirmed the strong interconnection between the two systems and lay the foundations for further studies that propose probiotic administration in order to decrease pulmonary exacerbation. Moreover, they noted clusters of bacteria, including potential pathogens such as *Enterococcus,* being present early in life in the gut and later in life in the respiratory tract, which highlights the potential interrelatedness of these two organ systems and their microbiota [19].

Hoen et al. analysed 120 faecal samples from 13 CF children, collected from birth to 34 months of age [43]. They noted a significant association between increased diversity of the gut microbiota and prolonged periods of health, delays in the time to initial *P. aeruginosa* colonization, and the first CF exacerbation. Moreover, they noted a reduction in two important gut colonizers, *Bacteroides* and *Bifidobacterium*, in stool samples prior to the first CF exacerbation and initial *P. aeruginosa* colonization, although this finding was non-significant [43]. Similarly, Antosca et al. examined the correlation between the composition of the stool microbiota and airway exacerbations in CF subjects, comparing stool samples from 21 CF infants and 409 controls [18]. During this study, they found a significant association between gut microbiome beta diversity and pulmonary exacerbations during the first year of life and confirmed the reduction in *Bacteroides* in CF infants as early as 6 weeks of life, a reduction persisting over the entire first year of life and confirmed in adulthood [18]. *Bacteroides* is a genus known for its immunomodulant role, demonstrated in vitro and in vivo. In vitro, the exposure of the apical face of polarized intestinal cell lines to *Bacteroides* species supernatants significantly reduces the production of Interleukin 8 (IL-8), suggesting a mechanism whereby changes in the intestinal microbiota may impact inflammation in CF [18]. A low proportion of *Bacteroides* has been associated with the risk of developing atopy and asthma [44,45], and *Bacteroides fragilis* in particular is known to be involved in modulating the Th type 1/2 (Th1/Th2) balance [46]. Decreased amounts of *Bifidobacterium* spp. are observed in the CF population, both in general and, most of all, after antimicrobial treatment, probably because of their high antimicrobial susceptibility, as explained above [21]. High bifidobacterial species richness is positively correlated with the maturation of the mucosal immune system. Conversely, an overall reduction in *Bifidobacteria* in children with CF may influence extra-intestinal disorders, such as respiratory inflammation and infection [47]. The imbalance in the gut microbiota, in particular, the reduction in immunomodulatory genera, may be a potential target in the development of probiotics dedicated to the CF population [48].

In terms of metabolite disruption, SCFAs, such as acetate, butyrate, propionate, and pantetheine, are known to be reduced in stool samples from CF patients as compared to healthy controls [20,37]. SCFAs are known to be involved in the promotion of differentiation of regulatory T cells [37] and in the regulation of inflammatory processes. In particular, SCFAs act to regulate several leukocyte functions, including the production of cytokines (TNF-α, IL-2, IL-6 and IL-10), eicosanoids, and chemokines (e.g., MCP-1 and CINC-2) [40]. Their reduction is likely the result of CF gut dysbioses, such as the decrease in *Bifidobacterium*, secondary to antibiotic use, or other butyrate-producing bacteria, such as *Eggerthella*, *Anaerostipes*, *Butyricicoccus*, and *Ruminococcus* [22]. These findings represent further confirmation of the close correlation between the gut and lungs and confirm the gut–lung axis theory, which has been previously acknowledged in the general population, in CF patients [49].

Overall, characteristics of gut composition in CF patients include reductions in immunomodulating genera and their metabolites. Although studies involving larger numbers are needed, evidence of a possible systemic pro-inflammatory effect and of a direct impact on the respiratory tract’s microbial composition exists. Those findings represent the rationale behind the potential use of dedicated probiotics in the CF population.

## 5. Gut Microbiota in CF: Possible Influence on Growth and Lung Function

Finally, several studies now address the systemic influence of the altered gut microbiota in patients suffering from CF, looking at its correlation with readings such as growth and lung function, which are known to be related to survival in this group [50]. Loman et al. reported a negative correlation between weight-for-length and a relative abundance of the *Staphylococcus* and *Faecalibacterium* species, while no correlation has been noted between the alpha diversity and any anthropometric measurement [12]. Hayden et al. identified an early, progressive faecal dysbiosis, distinguishing between infants with CF, a low length from infants with CF, and normal length [51]. This dysbiosis included altered abundances of taxa that perform important functions for gut health, nutrient harvest, and growth hormone signalling, including decreased *Bacteroidetes* and increased *Proteobacteria*. Coffey et al. investigated the composition and function of the bacterial communities inhabiting the intestines of children with CF and analysed their correlation with biomarkers of intestinal inflammation, growth, and lung function [37]. They demonstrated positive correlations between intestinal inflammatory markers, intestinal genera, and both growth z-scores and FEV1%. In particular, a relative abundance of *Ruminococcaceae* UCG 014 was positively correlated with BMI z-scores, and the *Ruminococcaceae* NK4A214 group was positively correlated with FEV1%. Intestinal inflammation was measured by faecal calprotectin levels and found to be significantly higher in the CF cohort compared to the healthy control, with a strong positive correlation between calprotectin and *Acidaminococcus* in CF [37]. An increased relative abundance of *Acidaminococcus* has been associated with lower future height z -cores in twin cohorts of children from Malawi and Bangladesh [52]. Furthermore, faecal calprotectin has a known negative correlation with height and weight z-scores among CF children [53].

Several studies on CF patients thus evidence a correlation between dysbiosis involving different genera and species that have a direct impact on inflammation, growth, and lung function, therefore confirming the hypothesis that the gut microbiota composition has effects that go well beyond the gut.

## 6. Conclusions

The gut environment in CF patients has unique features due to the characteristics of the disease, such as decreased bicarbonate secretion, increased luminal viscosity, and an acidic small intestinal environment, which, due to the treatment, includes regular antibiotic use or a high-energy and fat-dense diet. As a result, the gut microbial composition appears altered, with decreased richness and diversity, both of which are features seen in other inflammatory and systemic diseases. Moreover, the population of pro-inflammatory bacteria is higher, while immunomodulatory genera, such as *Bacteroides* and *Bifidobacterium*, are scarcer. The imbalanced gut microbial population has a potential role in the development of systemic inflammation and may influence clinical outcomes, such as respiratory exacerbations, spirometry results, and overall growth. Although a better understanding of the pathophysiology behind the gut–lung axis is needed, these findings support the rationale to consider gut microbiota manipulation as a possible intervention to regulate the severity and progression of the disease.

## Figures and Tables

**Table 1:** Evidence on factors influencing microbial acquisition and development.

Factors Involved	Reference	Study Evidence	Taxa Imbalance
Age	Loman et al. [12]	- Age as most predictive factor of overall faecal microbial composition in CF children - Solid food introduction and different substrate availability as main driver for the age-related changes	- ↑ *Blautia* and *Parabacteroides* positively correlated to age - ↑ *Bacteroides, Parabacteroides, Faecalibacterium, Butyricicoccus, Oscillibacter, Coprococcus, Blautia,* and other members of *Lachnospiraceae* after the introduction of solid food
CFTR dysfunction and CFTR modulators	Meeker et al. [13]	CFTR dysfunction actively modulates and selects gut microbiome in mice	
	Burke et al. [14]	No significant differences in species richness or microbial diversity between the CF cohort with class 1–3 mutations and other classes	- ↑ *Enterococcaceae* - ↓ *Ruminococcaceae* in CF patients with less severe mutations
	Schippa et al. [15]		- ↑ *Escherichia coli* and *Eubacterium biforme* - ↓ *Faecalibacterium prausnitzii*, *Bifidobacterium* spp., and *Eubacterium limosum* in patients with F508del and in more severe CF patients
	Ooi et al. [16]	No significant difference in alpha and beta diversities 6 months after starting ivacaftor	- ↑ *Akkermansia* 6 months after starting ivacaftor
	Ronan et al. [17]	No significant change in gut microbiota diversity and richness after a year of treatment with ivacaftor	
Delivery method	Loman et al. [12]	↑ alpha diversity in children born by C-section	- ↑ *Turicibacter* in children born by C-section, undetected in all vaginally born
	Antosca et al. [18]	Delivery mode not affecting significantly gut microbiota of CF infants	
Breastfeeding	Loman et al. [12]	Similar alpha and beta diversities in formula-fed vs. breastfed or mixed formula- and breastfed	- ↑ *Lactococcus* in children who were exclusively formula-fed
	Madan et al. [19]	- Non-significant overall diversity in gut microbial population between breastfed and formula-fed infants - Significant effect of breast milk exposure on respiratory tract microbial diversity	
Antibiotics use	Vernocchi et al. [20]	- No significant impact of single antibiotic therapy on the alpha diversity - Azithromycin plus aerosol antibiotic therapy worsened alpha diversity compared to healthy controls	- ↑ *Clostridium*, *Clostridium hiranosis*, *Eubacterium*, and *Faecalibacterium* in CF patients on chronic aerosol antibiotic therapy
	de Freitas et al. [21]		- ↓ *Bifidobacterium* in CF patients requiring antibiotic therapy compared to CF patients not requiring antibiotics
	Burke et al. [14]	Significant negative correlation between the number of IV antibiotic courses and gut microbiota diversity	- CF adults receiving the greatest exposure to IV antibiotics had ↑ *Firmicutes* and ↓ *Bacteroides* - ↓ *Bifidobacterium* and *Akkermansia* in CF adults who received macrolides
	Kristensen et al. [22]	Independent association between antibiotic treatment (mainly co-trimoxazole) and lower alpha diversity in CF infants	- Antibiotic use in CF infants associated with ↓ *Bifidobacterium* and Bacteroides and ↑ *Enterococcus*

↑, increased; ↓, decreased.

**Table 2:** Study evidence on gut–lung axis in CF children.

Gut-Lung Axys: Study Evidence in Children with Cystic Fibrosis		
Authors	Study Population	Study Evidence
Madan et al. [9]	7 CF patients from birth to 9-21 months of age	- Significant effects of breast milk exposure on respiratory tract diversity - Changes in diet results in altered respiratory microflora - Bacteria present early in life in the gut found later in life in the respiratory tract
Hoen et al. [43]	13 CF children from birth to 34 months of age	- Increased diversity of gut microbiota associated to prolonged periods of health, delays in the time to initial *P. aeruginosa* colonization and first CF exacerbation
Antosca et al. [18]	21 CF infants and 409 controls sampled between 6 weeks and 12 months of age	- Significant association between gut microbiome beta diversity and pulmonary exacerbations in the first year of life - Reduction of *Bacteroides* (immunomodulant genus) in CF infants as early as 6 weeks of life

## Data Availability

All data are included in the manuscript.
